# Diagnostic performance of GeneXpert in tuberculosis–HIV co–infected patients at Asella Teaching and Referral Hospital, Southeastern Ethiopia: A cross sectional study

**DOI:** 10.1371/journal.pone.0242205

**Published:** 2021-01-27

**Authors:** Abebe Sorsa, Muhammedawel Kaso

**Affiliations:** Arsi University College of Health Science and Referral Hospital, Asella, Ethiopia; The University of Georgia, UNITED STATES

## Abstract

**Background:**

GeneXpert is a new introduction in the diagnostic modality to fight tuberculosis (TB) among people living with HIV (PLHIV) under the program of intensified TB case finding. This study aimed to evaluate the diagnostic performance of GeneXpert under the program of intensified TB cases finding among PLHIV.

**Methods:**

Cross-sectional study was conducted by recruiting individuals attending an HIV clinic from February 2018 to January 2019. Data on clinical parameters were collected using a standardized tool. Two-morning sputum samples were collected and processed for smear microscopy and GeneXpert. SPSS 21 used for data analysis. Proportion, percentage, and mean with SD were used to describe variables. Univariate and multivariable logistic regressions were used to assess factors associated with the GeneXpert. Values for which the 95% CI interval not includes 1 and for which P<0.05 were considered significant.

**Result:**

A total of 384 presumptive TB-HIV co-infection cases were included, of which 166 (43%) were diagnosed to have TB. Fifty-four (32.5%) TB cases were smear AFB positive while 79 (47.7%) TB cases were GeneXpert positive. The GeneXpert detection rate was almost two-fold of that of smear microscopy and all smear positive TB cases were detected by GeneXpert. Moreover, GeneXpert was able to detect an additional third of TB confirmed cases among smear AFB negative cases. Advanced stage of the disease, high viral load and presence of anemia were significantly associated with TB. The WHO TB screening tool remained least sensitive with the lowest positive predictive value.

**Conclusion:**

GeneXpert demonstrated two-fold case detection rate compared to the sputum smear microscopy and additional third TB case detection rate among smear AFB negative cases. Clinical screening tool for evaluation of TB-HIV co-infection showed poor performance in TB case notification.

## Background

Tuberculosis (TB) is among the most widespread and serious of all human infectious diseases. Owing to widespread poverty, inequity and conflict, suboptimal health services in many countries and the impact of HIV/AIDS pandemic, there are more cases of TB today than at any time previously in human history. Close to 95% of all cases, and 98% of deaths due to TB, occur in tropical countries [1]. TB is the most frequent life-threatening opportunistic infection and a leading cause of death among PLHIV. HIV-positive people with latent TB infection have a 10% annual and 50% lifetime risk of developing active TB disease [2]. Similarly, about 79% of HIV-positive clients were screened for TB; of these 11% were found to have active TB. World Health Organization (WHO) recently introduced the algorithm of intensified TB case finding among PLHIV [3–5]. WHO recommends that TB case findings should be intensified in all human immunodeficiency virus (HIV) testing and counseling services for HIV-positive clients by using a set of simple questions for early identification of TB suspects [6–8]. Diagnosis of TB is challenging in HIV-positive individuals, especially when the stage of the disease is advanced. Standard TB diagnostic approaches and clinical algorithms should be followed to guide the diagnosis of TB in PLHIV. Sputum smear Acid fast bacilli (AFB)- microscopy has been in clinical use for decades for the diagnosis of TB, and sensitivity varies between 30–70%. In individuals co-infected with HIV, it is varies between 20% and 50% [9–11]. The development of GeneXpert represents a paradigm shift in the diagnosis of TB and drug-resistant. It simplifies molecular testing by fully integrating and automating the three processes required for real-time PCR-based molecular testing (that is, specimen preparation, amplification, and detection) and it detects both live and dead bacteria [9]. GeneXpert detects *M*. *tuberculosis* as well as mutation that confer rifampicin resistance using three specific primers and five unique molecular probes to ensure a high degree of specificity [10]. The assay provides results directly from sputum in less than 2 hours [9, 10]. The sensitivity of the GeneXpert test is much better than smear microscopy test and comparable to solid culture. Previous studies reported the sensitivity and specificity of GeneXpert to be 72–77% and 99% in smear-negative adults and 98–99% and 99–100% in smear-positive adults, respectively. The negative predictive value (NPV) was 90.6%, and the positive predictive value (PPV) was 100%. Limit of detection is 5 genome copies of purified DNA per reaction or 131 colony forming units/ mL in *M*. *tuberculosis* spiked sputum in comparison of identification of TB bacilli by microscopic examination requires at least 10,000 bacilli per mL of sputum [9–16]. In spite of early initiation of highly active antiretroviral therapy (HAART), TB remains a significant cause of morbidity and mortality among people living with HIV infection in Sub-Saharan Africa [10]. In Ethiopia, even though more than 400,000 HIV-positive people were enrolled in chronic HIV care with an annual enrolment of around 30,000 newly diagnosed HIV positives in 2016, only 0.4% of them were diagnosed to have active TB while only 40% of the newly enrolled HIV positives were reported to access preventive therapy as per national recommendation [15–17]. This implies a large segment of HIV-positive TB cases did not reach care. Prompt and accurate diagnosis of TB and timely initiation of appropriate treatment play a pivotal role in reducing the suffering of the patients and contributes a remarkable role in halting TB transmission [5, 6].

The national TB-HIV collaborative work emphasized on three I’s which include **I**ntensive case findings, **I**NH Preventive Therapy, and **I**nfection control to mitigate the morbidity and mortality associated with TB-HIV co-infection. The Ethiopia national TB prevention and control program stressed the intensified TB case-finding approach by devising simple screening symptoms that include any one of the following (current cough, weight loss, and fever). Limited information exists in Ethiopia regarding the performance of GeneXpert under the program for intensified TB case finding among PLHIV [5]. Therefore, this study was aimed to evaluate the diagnostic performance of GeneXpert in TB- HIV co–infected patients at Asella Teaching and Referral Hospital in Southeastern Ethiopia.

## Methods and material

### Ethical consideration

Research proposal was initially submitted to Arsi University College of Health Science Research and Ethical Review Board (IRB) and ethical approval was obtained to pursue the study with reference number of AU_TB_10/2017. After briefing about the purpose and benefit of the study, a consent form was signed by the patient before commencing data collection. Confidentiality of information about the patients’ medical problem was secured with maximum effort and was not exposed to any other third party.

### Study setting

The study was conducted at Arsi University Asella Teaching and Referral Hospital one of the referral hospitals in the South east part of the country, delivering service for close to 3–4 million people. The region is one of the highest TB prevalent area, and Asella Teaching and Referral Hospital has been reporting more than 1000 annual TB cases [7, 18]. Hence, the Hospital was given priority during the initial phase of GeneXpert service roll out program and equipped with the machine in 2016 [7, 18]. During the first three years, the diagnostic service of this new technology was indicated for special group patients which include TB-HIV co-infection, childhood TB and suspected MDR-TB but since mid of 2019, GeneXpert became the diagnostic of choice for all suspected TB cases despite frequent interruption of the service because of shortage of cartridge [7]. The Hospital has been also one of the treatment centers for PLHIV for more than a decade and at the study time there were about 3560 PLHIV on follow-up at the infectious disease clinic of the hospital as reported from HIV care, treatment and control registry book.

### Study design

We conducted a Hospital based cross-sectional study. The source populations were individuals attending an HIV clinic from February 2018 to January 2019 at Asella Teaching and Referral Hospital.

#### Inclusion criteria

Patients fulfilling the revised national/WHO screening tool for TB assessment among PLHIV were included [5, 7]. As per the National/WHO TB screening tool among PLHIV, we included all patients who fulfilled one of these clinical features (current cough, fever or weight loss) during their regular follow up visit.

#### Exclusion criteria

Those patients already diagnosed to have TB and receiving anti-TB medications at the time of the survey and patients fulfill clinical definition but who couldn’t give sputum for examination were excluded.

### Data collection tools and procedures

Patients who fulfilled inclusion criteria were interviewed for socio-demographic variables and evaluated for clinical parameters. Data were annexed to the structured check list tool. Then patients were asked to provide two morning spot sputum specimens (>2mL each) for AFB microscopy and GeneXpert with 1 hour apart. Sputum samples were not induced, but collection was directly observed by one investigator. Smear microscopy was performed by two experienced laboratory technologists throughout the study period. Direct smear microscopy for AFB was performed using Ziehl- Neelsen staining using LED microscopy as per SOP [7, 18]. The test results were reported in accordance with WHO/International Union Against TB and Lung Diseases recommendations [2]. Whenever there were discordant smear AFB reports, the findings were correlated with other clinical findings and GeneXpert reports results to make a final decision.

After sputum sample smear microscopy was used, the remainder of the sputum specimen was tested for GeneXpert v 4.3 (Cepheid, Sunnyvale, CA) according to manufacturer’s instructions and national implementation Guideline for GeneXpert MTB/RIF Assay in Ethiopia [5]. Interpretation, Case definitions, Registration, and Management of TB and RR cases based on Xpert MTB/RIF test results were per the WHO (2013) update on “definitions and reporting framework for Tuberculosis” [5]. The laboratory technologists who performed the two tests (smear AFB microscopy and GeneXpert) were independent and were blinded for the results of each test.

### Data processing and analysis

Data were coded and entered to EPI-info version7.1.1 for clean-up and then exported to SPSS 21for further analysis. Descriptive statistics like frequency, percentage, proportion, and mean with standard deviation (SD), median with interquartile range (IQR) were used. Univariate logistic regression was used to assess factors associated with the yield of sputum microscopy and GeneXpert. Variables with p-value of <0.2 were further analyzed using multivariable regression. Frequencies were calculated for categorical variables and mean with SD and median with IQR were calculated for continuous variables. The factors were considered to be associated when p value was <0.05 and or when 95% confidence interval did not include 1

### Operational definition [6–8, 18]

#### Compatible chest X-ray with pulmonary TB

Chest X-ray report with any of the following finding; bronchiectasis, consolidation, patchy, cavitary, and miliary TB.

#### TB cases

All forms of TB which include both pulmonary and disseminated TB.

#### Pulmonary TB

A form of TB contained only to the lung.

#### Disseminated TB

A clinical spectrum of TB involving lung and one or more of other organ systems.

#### Presumptive TB case

Any patient who fulfills the national screening for clinical symptoms of TB.

#### Probable TB cases

If patient met two or more of the following criteria; history of tuberculous contact, clinical features suggestive of TB, reactive tuberculin, skin test >10 mm, or radiographic findings compatible with TB (miliary disease, cavitary lesions, hilar lymphadenopathy, or primary complex), no response to broad spectrum antibiotic trial.

## Results

A total of 384 presumptive TB-HIV co-infection cases were enrolled. Males accounted for 199 (51.9%) and the mean age of patient was 37±12.4 years. Most patients were WHO HIV/AIDS clinical stage II and stage III, each accounting for 119 (30.9%) and 130 (33.9%), respectively. The median CD_4 count_ at the time of TB consideration was 256 cell/ml with IQR of (129–452), and about 104 (38.2%) had CD_4_ cell count of less than 200 cell/ml. Of those patients who had HIV viral load determination during the last six months at the time of TB screening, 134 (78.8%) had viral suppression less than 1000copies/ml. Cough as positive screening symptoms was reported by 372 (97.1%) patients while fever was reported by 298 (77.8%) patients (Table 1). All patients have been on antiretroviral therapy (ART) with duration of ART treatment ranging from 0–132 months with a median duration of 52 months. A total of 166 (43.2%) TB cases were diagnosed during the study period and 79 (49.0%) TB cases were bacteriologically confirmed either with GeneXpert or sputum AFB.

**Table 1:** Demographic and some clinical data of presumptive TB-HIV co-infection patients among people living with HIV, Asella Teaching and Referral Hospital 2019. (n = 384).

Characteristics			N (%)
Sex	Male		199(51.8)
	Female		185(48.2)
Age category(years)	= <18 years		45(11.7)
	18–60 years		324(84.4)
Clinical characteristics at base line	Cough		372(97.1)
	Fever		298(77,8)
	Weight loss		257(67.1)
	Previous TB treatment		84(21.9)
	INH prophylaxis		161(42.0)
	CPT prophylaxis		302(78.9)
	WHO clinical stages	III	53(13.8)
		I	119(30.9)
		II	130(33.9)
Laboratory	Hematocrit	Normal	224(59.4)
		Anemia	153(40.6)
	CD4 count	<200cell/ml	104(38.2)
		> = 200cell/ml	168(61.8)
	HIV viral load	<1000copy/ml	39(48.1)
		> = 1000copy/ml	42(51.9)

### Concordance of GeneXpert assay and sputum acid-fast smear (AFB)

The case notification rates of both sputum AFB and GeneXpert was evaluated among presumptive TB cases and the case detection rate of sputum smear AFB was 51 (13.3%) while that of GeneXpert was almost two-fold higher, 79(20.6%). GeneXpert increased the TB detection rate by 25 cases (31.6%) compared with sputum smear AFB. The agreement between the two tests was found to be substantial with *kappa* value of 0.74.

### Sensitivity, specificity, positive predictive value (PPV) and negative predictive value (NPV) of national/WHO symptom screening tool of TB among PLHIV

The sensitivity, specificity, PPV, and NPV of national/WHO screening tool of TB among PLHIV patients varied based on whether the tools were used singly, or in combination; if cough used as sole screening tool the sensitivity, specificity, PPV and NPV were found to be 3.6%, 100%, 21.2% and 100% respectively. When cough and fever were combined as screening tool, the sensitivity, specificity, PPV, and NPV became 25.5.%, 84.5.5%, 22.6%, and 86.8% respectively, and if the three screening symptoms were positive, the sensitivity, specificity, PPV and NPV showed 44.4%, 60.0%, 21.8% and 80.4% respectively (Table 2).

**Table 2:** Sensitivity, specificity, PPV and NPV TB screening tool among PLHIV, Asella Teaching and Referral Hospital 2019.

Screening tools	GeneXpert confirmed TB cases, N = 79			
	Sensitivity	Specificity	PPV	NPV
Cough +fever+ weight loss	44.4%	60.0%	21.8%	80.4%
Cough + fever	25.5%	84.5%	22.6%	86.8%
Cough +weight loss	34.5%	66.7%	20.7%	80.2%
Cough only	3.6%	100%	21.2%	100%

NPV-negative predictive value, PLHIV-people living with HIV, PPV-positive predictive value

### Clinical factors associated with results of GeneXpert and smear AFB

Using univariate analysis, body mass index (BMI), duration of cough, chronic medical illness, previous TB treatment, isoniazid preventive therapy (IPT), WHO clinical staging of HIV/AIDS were positively associated with the result of both sputum AFB microscopy and GeneXpert (Table 3). However, using multivariable logistic regression, longer duration of cough, advanced stage of the disease, presence of anemia, and high HIV viral load were significantly associated with positive sputum AFB microscopy and GeneXpert results (Table 4). Lower BMI was associated with positive sputum AFB microscopy but not statistically associated with positive GeneXpert results.

**Table 3:** Clinical and laboratory findings among patients with sputum AFB microscopy and GeneXpert positive results, Asella Teaching and Referral Hospital 2019.

Variables		Smear AFB positive TB cases, N = 54		GeneXpert positive = 79	
		Number (%)	p-value	Number (%)	P value
Duration of cough (in weeks)	<2	16(26.0)	0.02	14(17.7)	0.03
	≥2	38(74.0)		65(82.3)	
Previous TB treatment	Yes	17(31.5%)	0.05	22(27.8)	0.17
	No	37(68.5%)		57(72.2)	
Chronic medical illness	Yes	31(57.4%)	0.03	31(39.2)	0.001
	No	23(42.6)		48(60.8)	
Weight loss	Yes	29(53.7)	0.37	43(54.4)	0.58
	No	26(46.3)		36(45.6)	
IPT*	Yes	11(18.7)	<0.001	17(22.7)	<0.001
	No	43(81.3)		62(77.2)	
WHO* clinical staging of HIV/AIDS	III	40(74.1)	0.004	43(62.3)	0.18
	IV	14(25.9)		36(37.7)	
Anemia	Yes	45(83.3)	<0.001	62(78.5)	0.001
	No	9(16.7)		17(21.5)	
CD_4_ count(cell/ml)	<200	26(48.4)	0.21	67(85)	<0.001
	≥200	28(51.6)		12(15)	
HIV Viral load(copy/ml)	<1000	13(25))	0.001	60(75.7)	0.013
	≥1000	41(75.0)		19(24.3)	
BMI (kg/m^2^)	<18.5	33(61.0)	0.19	47(60)	0.17
	≥18.5	21(39.0)		32(40)	

ESR = erythrocyte sedimentation rate, IPT = Isoniazid preventive therapy, BMI = body mass index, WHO = world health organization, hr = hours, AFB = Acid fast bacilli, AIDS = acquired immunodeficiency syndrome, HIV-human immunodeficiency virus, TB = tuberculosis

**Table 4:** Univariate and multivariate logistic regression assessing factors associated with the result of GeneXpert and smear AFB, Asella Teaching and Referral Hospital 2019.

		Smear positive TB cases, N = 54		GeneXpert positive, N = 79	
		COR, 95%C	AOR, 95%CI	COR (95%CI)	AOR (95%CI)
BMI* (kg/m^2^)	<18.5	1	1	1	1
	≥18.5	1.97(1.27–4.44)	2.00(1.07–3.70)	1.74(1.14–2.65)	1.051(0.67–3.70)
Duration of cough (in weeks)	<2	1	1	1	1
	***≥2***	1.28(1.16–2.47)	2.55(1.96–5.53)	1.31(0.67–2.57)	2.39(1.28–4.48)
Previous TB treatment	Yes	2.08(1.21–3.58)	1.22(0.76–1.95)	1.78(1.05–3.02	1.44(0.89–2.31)
	No	1		1	1
Chronic medical illness	Yes	1.95(1.13–4.61)	2.42(1.24–5.73)	3.63(1.33–8.08)	2.688(1.41–6.92)
	No	1	1	1	1
IPT*	Yes	1	1	1	1
	No	5.7(3.2–10.2)	7.5(4.5–12.6)	3.34(1.62–6.90)	3.21 (1.79–5.75)
WHO* clinical staging of AIDS	III	1	`1	1	1
	IV	10.21(1.35–77.4)	6.32(1.35–35.43)	10(1.35–100.00	25(3.33–90.91)
Anemia	Yes	4.33(2.05–9.18)	4.71(2.10–10.58)	4.88(2.22–10.69)	3.528(2.10–6.31)
	No	1	1	1	1
CD_4_ count (cell/ml)	<200	1.48(0.69–3.17)	1.05(0.45–2.48)	2.25(0.69–7.34)	1.73(0.63–4.80)
	> = 200	1	1	1	1
HIV Viral load(copy/ml)	<1000	1	1	1	1
	≥1000	0.67(0.24–1.87)	1.13(0.31–4.15)	2.67(1.08–6.583)	1.87(1.21–4.085)

ESR = erythrocyte sedimentation rate, IPT = Isoniazid preventive therapy, BMI = body mass index, WHO = world health organization, hr = hours, AFB = Acid fast bacilli, AIDS = acquired immunodeficiency syndrome, HIV-human immunodeficiency virus, TB = tuberculosis

Patients who have never taken IPT during their course of HIV illness were at significant risk of developing TB, inferring the preventive efficacy of IPT to be 78%.

### Laboratory finding associated with yield of GeneXpert and smear AFB

Using univariate logistic regression: anemia, increases in erythrocyte sedimentation rate (ESR), CD4 cell count, HIV Viral load were associated with positive results of GeneXpert and smear AFB. However, using multiple logistic regression, anemia and high HIV viral load were significantly associated with positive sputum AFB microscopy and GeneXpert (Table 4).

## Discussion

Our study focused on the role of GeneXpert in TB case notification under the program of WHO intensified TB case finding among PLHIV. GeneXper is a relatively a new diagnostic modality in the battle to combat TB as a global public health problem, and is believed that it is to improve bacteriology confirmed TB cases with shorter turnaround time as shown in previous studies [1, 4]. In this study, the case detection rate of GeneXpert was compared with the conventional smear microscopy and revealed that it showed almost two-fold case detection rate. All patients who turned positive for smear AFB were also positive for GeneXpert, implying that it can replace the conventional smear AFB microscopy in the clinical care of TB [9, 12, 13]. GeneXpert detected additional 31.6% among smear AFB negative cases which is congruent with the finding of the study from north-western part of Ethiopia and other multicenter studies [1, 4, 7, 13]. However, the study finding reported by Habte et al indicated additional TB case detection of GeneXpert among smear AFB negative cases was 64.3% [19], which was higher than our finding while the report from Cochrane review and meta-analysis by Steingart and his collogues, and studies from other areas were in the range of quarter which were lower than our finding [5, 6, 9, 14, 17, 20–25]. The difference could be related with enrollment criteria of study participants in which our study was used the revised National/WHO screening tool protocol for TB-HIV co-infection among PLHIV which tended to indicate very low positive predictive value.

The sensitivity, specificity, PPV, and NPV of the revised national/WHO screening tool were evaluated in our study [6–8, 18]. Accordingly, if a cough is used as a sole screening tool of TB, the sensitivity is very poor implying that patients who were coughing were not actually having the disease. However, the specificity of these screening tools was very good, indicating that most patients whowere not coughing or having fever were less likely to have TB. The good specificity of the screening tool for TB is within the recommended minimum proposed specificity requirement of 70% proposed by the WHO for a TB triage test [4, 8]. This strategy has been limited in part by a low sensitivity of symptom screening leading to a large number of patients were in need of tests even though the results in most cases turned to be negative resulting in resource exploitation and manpower burden. The argument here is that it is more important not to miss even a single case of TB at an expense of resource and manpower exploitation [8].

In our study, patients who were having anemia were found to be highly likely of having TB which could be explained by the actual presence of chronic illness which actually causes anemia of chronic illness. Similar findings were reported by Temesgen et al and other reports [3, 4].

Our study also concluded that HIV viral load above 1000copy/ml was significantly associated with the occurrence of TB while CD4 has no association with TB diagnosis, implying that HIV viral load was more reliable indicator of predicting the degree of HIV/AIDS disease status and opportunistic infection [19, 20, 26–28].

Other clinical factors like advanced WHO clinical stage of HIV/AIDS was significantly associated with the development of TB, implicating that the higher the stage of the disease, the weaker the immune system to contain the bacilli leading to flare-up of the disease. Consistent findings were reported from other studies [15, 29–31].

One of the strategies to prevent the development of TB among PLHIV is the provision of isoniazid preventive therapy (IPT). In our report, among the presumptive TB cases, a total of 161(42%) PLHIV took IPT. The risk of developing TB among patients who didn’t take IPT was five-fold which is comparable with reports from other studies [15, 32].

In conclusion, the introduction of GeneXpert significantly improved the case detection of TB compared to the conventional smear microscopy and it additionally detected third TB cases among smear AFB negative presumptive HIV-TB co-infection. Advanced stage of the disease and poorly suppressed viral load were the two independent factors predicting the results of GeneXpert. Using symptom based screening of TB-HIV co-infection showed poor validity in TB case notification.

## Supporting information

S1 File (BIN)

S1 Data (XLS)
